# Assessment of Demineralization Inhibition Effects of Dentin Desensitizers Using Swept-Source Optical Coherence Tomography

**DOI:** 10.3390/ma14081876

**Published:** 2021-04-09

**Authors:** Kumiko Matsuzaki, Yasushi Shimada, Yasuo Shinno, Serina Ono, Kozo Yamaji, Naoko Ohara, Alireza Sadr, Yasunori Sumi, Junji Tagami, Masahiro Yoshiyama

**Affiliations:** 1 Department of Operative Dentistry, Field of Study of Biofunctional Recovery and Reconstruction, Okayama University Graduate School of Medicine, Dentistry and Pharmaceutical Sciences, Okayama 700-8558, Japan; shimada.ope@okayama-u.ac.jp (Y.S.); shin-no@md.okayama-u.ac.jp (Y.S.); de421008@s.okayama-u.ac.jp (S.O.); yamaji@md.okayama-u.ac.jp (K.Y.); ohara-n@cc.okayama-u.ac.jp (N.O.); yoshiyam@md.okayama-u.ac.jp (M.Y.); 2 Biomimetics Biomaterials Biophotonics Biomechanics & Technology Laboratory, Department of Restorative Dentistry, University of Washington, Seattle, WA 98195-7456, USA; arsadr@uw.edu; 3 Center of Advanced Medicine for Dental and Oral Diseases, Department for Advanced Dental Research, National Center for Geriatrics and Ger Ontology, Aichi 474-8511, Japan; yasusumi@ncgg.go.jp; 4 Department of Cariology and Operative Dentistry, Graduate School of Medical and Dental Sciences, Tokyo Medical and Dental University, Tokyo 113-8549, Japan; tagami.ope@tmd.ac.jp

**Keywords:** SS-OCT, dentin desensitizer, dentin demineralization

## Abstract

The purpose of this study was to evaluate the mechanism of action and the inhibiting effects of two types of desensitizers against dentin demineralization using pre-demineralized hypersensitivity tooth model in vitro. In this study, we confirmed that a hypersensitivity tooth model from our preliminary experiment could be prepared by immersing dentin discs in an acetic acid-based solution with pH 5.0 for three days. Dentin discs with three days of demineralization were prepared and applied by one of the desensitizers containing calcium fluoro-alumino-silicate glass (Nanoseal, NS) or fluoro-zinc-silicate glass (Caredyne Shield, CS), followed by an additional three days of demineralization. Dentin discs for three days of demineralization (de3) and six days of demineralization (de6) without the desensitizers were also prepared. The dentin discs after the experimental protocol were scanned using swept-source optical coherence tomography (SS-OCT) to image the cross-sectional (2D) view of the samples and evaluate the SS-OCT signal. The signal intensity profiles of SS-OCT from the region of interest of 300, 500, and 700 µm in depth were obtained to calculate the integrated signal intensity and signal attenuation coefficient. The morphological differences and remaining chemical elements of the dentin discs were also analyzed using scanning electron microscopy and energy-dispersive X-ray spectroscopy. SS-OCT images of CS and NS groups showed no obvious differences between the groups. However, SS-OCT signal profiles for both the CS and NS groups showed smaller attenuation coefficients and larger integrated signal intensities than those of the de6 group. Reactional deposits of the desensitizers even after the additional three days of demineralization were observed on the dentin surface in NS group, whereas remnants containing Zn were detected within the dentinal tubules in CS group. Consequently, both CS and NS groups showed inhibition effects against the additional three days of demineralization in this study. Our findings demonstrate that SS-OCT signal analysis can be used to monitor the dentin demineralization and inhibition effects of desensitizers against dentin demineralization in vitro.

## 1. Introduction

Dentin hypersensitivity (DH) is a dental condition characterized by localized sharp pain arising in response to external stimuli, typically thermal, tactile, evaporative, osmotic, or chemical stress, without any other dental defects or disease [1,2,3,4,5,6]. The prevalence of DH varies from 3% to 98% for different evaluation methods and patient populations [7]. Although all age groups of patients are affected by DH, it frequently occurs in patients between 30 and 40 years old [8]. Erosion has been considered the main etiological factor for DH, because it is capable of opening and enlarging the dentinal tubules to allow for fluid to flow through the tubules [9,10,11]. Therefore, one of the main strategies for DH treatment involves depolarization of the nerve or occlusion of the dentinal tubules [12].

Several desensitizers have been introduced for treatment of DH to occlude dentinal tubules and reduce dentin permeability. The dentin desensitizer Nanoseal (Nippon Shika Yakuhin, Yamaguchi, Japan) is composed of a liquid (A), formed by dispersing fine calcium fluoro-alumino-silicate glass particles, and phosphoric acid aqueous liquid (B). The chemical composition of Nanoseal is similar to that of silicate cement, setting with initial acid–base reaction to form a less soluble phosphate salt. It also interacts with the inorganic components of dentin to form nano-sized deposits on the surface. DH can be suppressed by sealing the dentinal tubules with a nanoparticle-deposited layer, which provides resistance to demineralization [13,14,15,16].

Caredyne Shield (GC, Tokyo, Japan) is a tooth surface-coating material containing fluoro-zinc-silicate glass, which provides antibacterial properties [17], prevents dentin demineralization [18,19], and inhibits matrix metalloproteinase (MMP) activity [20,21]. Nanoparticle-sized deposits are also formed in this material with a thickness of approximately 1–2 µm on the dentin surface [17]. Similar to Nanoseal, this layer has acid resistant effect, which leads to the prevention of dentin demineralization. Considering the inorganic component of these materials, their potential to prevent dentin demineralization, along with their desensitizing effects, would be of interest to manage DH.

Restorative resin composites can be used to seal the hypersensitivity tooth surface if it is accompanied with tooth loss. The properties of restorative composites are influenced by resin matrix, reinforced fillers, silane coupling agents, initiator-activator systems, and inhibitors [22]. The mechanical behavior of resin composites is mainly evaluated by static or dynamic compression tests [23]. However, it is difficult to evaluate the demineralization inhibition effects of dentin desensitizers. Some authors have evaluated mineral loss and lesion depth using micro-focused X-ray computed tomography or a transverse micro radiography [14,16,17]. In most cases, dentin desensitizers are evaluated by observing dentinal tubules using (Scanning Electron Microscope) SEM [1,2,3,4,5,24].

Optical coherence tomography (OCT) is an interferometric technique that can create cross-sectional images of biological structures [25]. OCT images can differentiate the optical properties of various tissues, including the effects of optical absorption and scattering. Recently, OCT has been used to monitor the demineralization of enamel and dentin [26,27]. Amaechi et al. measured the percent of reflectivity loss because of demineralization on dentin surfaces and showed that this correlated well with the mineral loss from microradiography [28]. Sowa et al. reported that the attenuation coefficient of the OCT signal intensity increased after the demineralization of tooth [29]. OCT signal intensity and attenuation patterns of OCT images are influenced by the presence of demineralization and have been used as objective parameters to distinguish the demineralization of enamel and dentin [30]. The images and numerical values from non-destructive examination using OCT shows the potential for the diagnosis of DH that cannot be diagnosed by X-ray examinations.

The purpose of this study was to evaluate the mechanism of actions and the inhibiting effects of two types of desensitizers against dentin demineralization using pre-demineralized hypersensitivity tooth model in vitro. Swept-source optical coherence tomography (SS-OCT) imaging and signal analysis of the pre-demineralized dentin surface were performed with the application of desensitizers. For the confirmation study, demineralized specimens with the application of desensitizers were observed morphologically using SEM, and the remaining chemical elements were analyzed using energy-dispersive X-ray spectroscopy (EDX).

## 2. Materials and Methods

### 2.1. Materials

Two commercially available desensitizers (Table 1), Caredyne Shield and Nanoseal, were used as the experiment materials to compare the effect against dentin demineralization in vitro. A total of 30 caries-free human molars preserved in saline at 4 °C were used in this study. The usage of the extracted teeth was approved by the Ethics Committee of Okayama University (approval number 189). The crown portions of the teeth were sectioned horizontally using a low-speed diamond saw (IsoMet low speed saw, Buehler, Lake Bluff, IL, USA) under running water to obtain 30 tooth discs with a flat dentin surface parallel to the occlusal plane. The dentin surfaces were ground with #600 grit silicon carbide paper under running water to prepare the standard smear layer.

### 2.2. SS-OCT Analysis

Twenty-five dentin discs were selected from the 30 discs for SS-OCT analysis and the remaining five dentin discs were used for SEM analysis. The 25 discs were further divided into five groups of five samples each to allocate the following experimental protocol:

Group 1: sound dentin without any treatment as a control (sound).
Group 2: demineralized dentin for three days (de3).
Group 3: demineralized dentin for six days (de6).
Group 4: demineralized dentin for six days with Caredyne Shield. Caredyne Shield was applied to the demineralized dentin after three days of demineralization, and the surface was further demineralized for three days (CS).
Group 5: demineralized dentin for six days with Nanoseal. Nanoseal was applied to the demineralized dentin after three days of demineralization, and the surface was further demineralized for three days (NS).

The pulp side of all the dentin surfaces of Groups 2, 3, 4, and 5 were covered with nail varnish, and the occlusal dentin surfaces were exposed to create artificial demineralization. The specimen discs were then immersed in the demineralization solution containing 2.2 mM CaCl_2_, 2.2 mM Na_2_HPO_4_, and 50 mM CH_3_COOH (adjusted to pH 5.0 with 1 M KOH) at 37 °C for the experimental periods [31]. In this study, a demineralization period of three days was employed to create artificial hypersensitivity tooth model with the dentin surface, removing the smear layer and opening the dentinal tubules [11,32]. The degree of demineralization for this hypersensitivity tooth model was confirmed by our preliminary experiment.

**Figure 1:**
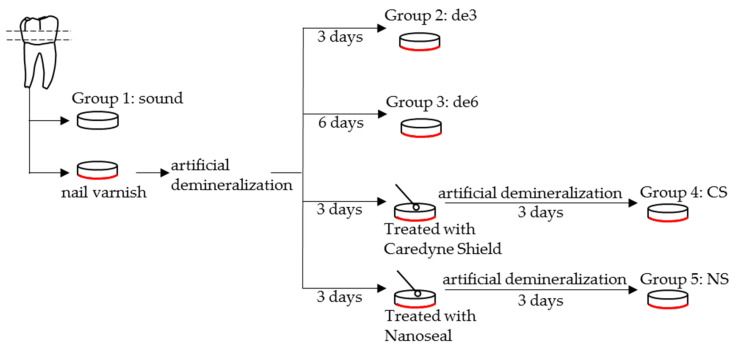
Schematic illustration of the dentin specimen preparation for swept-source optical coherence tomography (SS-OCT) and SEM analysis. The 25 dentin discs were divided into five groups of five samples each. An acetic acid solution with pH 5.0 was employed as the artificial demineralization solution, and the solution was replaced every day during the process.

In Groups 4 and 5, one of the desensitizing materials of Caredyne Shield or Nanoseal was applied to the dentin window after the three days of demineralization. The samples were again immersed in the demineralization solution for three days (Figure 1). The demineralization solution of all specimens was replaced every day.

Following each treatment protocol for the experimental groups, the dentin surfaces were subjected to SS-OCT scanning to obtain a cross-sectional (2D) image of the dentin and SS-OCT signal. The SS-OCT system used in this study was a frequency-domain OCT system (IVS-2000, Santec, Komaki, Japan). The central wavelength was 1310 nm, with sweeping in the wavelength range from 1260 to 1360 nm at a 20 kHz sweep rate. The scanning light was projected onto the observation sites across the area of interest using a handheld probe. The axial resolution of the device was 12 µm in air and 8 µm in dentin, assuming a refractive index of approximately 1.63 [33]. The lateral resolution of 17 µm was determined by the objective lens of the probe. A 2000 × 1024 pixel image was obtained in real time and processed in a few hundred milliseconds.

Five locations were randomly selected from each of the samples as the observation sites, where SS-OCT scanning was performed. As a result, 25 images in total were observed for each group. A signal intensity profile in dB unit for each SS-OCT image was obtained to calculate the values of maximum signal intensity, integrated signal intensity, and signal attenuation coefficient.

For the maximum signal intensity, the signal intensity profile up to the depth for 800 µm was employed from the SS-OCT image and averaged, where the value was calculated.

**Figure 2:**
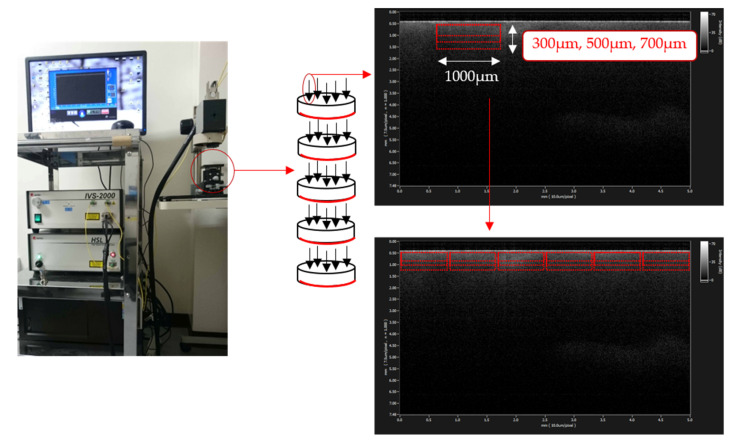
Setting the region of interest (ROI). Five locations were randomly selected on each of the five dentin discs for 25 points per group. A ROI width of 1000 µm × optical depth of 300, 500, or 700 µm from the surface of the specimen discs was set on the obtained SS-OCT images. The six areas of ROI at each depth were set in one image. The integrated signal intensity and signal attenuation coefficient in these ROIs were calculated.

For the integrated signal intensity and signal attenuation coefficient, a region of interest (ROI) with a width of 1000 µm and optical depth of 300, 500, or 700 µm from the surface of the specimen discs was set on the obtained SS-OCT images, avoiding the high Fresnel reflection of the outermost surface layer. Six areas of ROI were selected from one image (n = 150, Figure 2). A signal intensity profile of SS-OCT for each ROI was obtained to calculate the integrated signal intensities using image analysis software (Image J, NIH, version 1.47n, Bethesda, MD, USA) as well as signal attenuation coefficient (µt) using the equation derived from the Beer–Lambert law with the following formula [34,35,36]:

*I*(z) = ce^−2µz^ (1)

where *I* is the reflectivity signal intensity in (dB), c is a constant, and z is the depth variable, which has a factor of 2. The µt was calculated using linear least-squares regression to fit the natural log of the average OCT profiles obtained from the ROI.

(2) $$\mu_{t}\propto -\frac{\ln I(z)}{2z}$$

### 2.3. SEM/EDX Analysis

The remaining five dentin discs of the 30 discs were used for SEM study. A deep slit on the pulp side was initially prepared with a diamond bur to facilitate subsequent fracture of the dentin surface. The area other than the occlusal dentin surface was protected with a nail varnish to prevent the artificial demineralization solution from penetrating through the pulpal side. The dentin surfaces were allocated to one of the five groups and treated in the same manner as the SS-OCT specimens. After careful removal of nail varnish, the specimens were rinsed with deionized water for 10 s and dehydrated using ascending grades of ethanol up to 100%. The specimens were then immersed in t-butyl alcohol, critical point dried, and were gently split with a scalpel blade to provide a sagittal view of the treated dentin. The specimens were fixed on stubs with double-sided adhesive carbon tape and were coated with gold in a vacuum-metalizing machine. Both the part of the treated surfaces as well as the sagittally fractured surface of each tooth were examined using SEM (S-4800, Hitachi, Tokyo, Japan) operating at 5.0 kV [3,37,38]. To characterize the reactional products formed by the desensitizers, EDX (EDAX Genesis, Hitachi, Tokyo, Japan) was used to detect the chemical elements within the dentin tubules and on the dentin surface. The respective analysis was performed from the sagittally fractured surface. Images of the specimens were obtained at 15 kV at a magnification of 3000×.

### 2.4. Statistical Analysis

The integrated signal intensities and µt values were statistically analyzed using one-way ANOVA with Tukey’s post hoc between the groups. All of the statistical analyses were performed at a 95% level of confidence using JMP 12 software (SAS Institute, version12, Cary, NC, USA). Values of *p* < 0.05 were considered significant.

## 3. Results

### 3.1. SS-OCT Analysis

**Figure 3:**
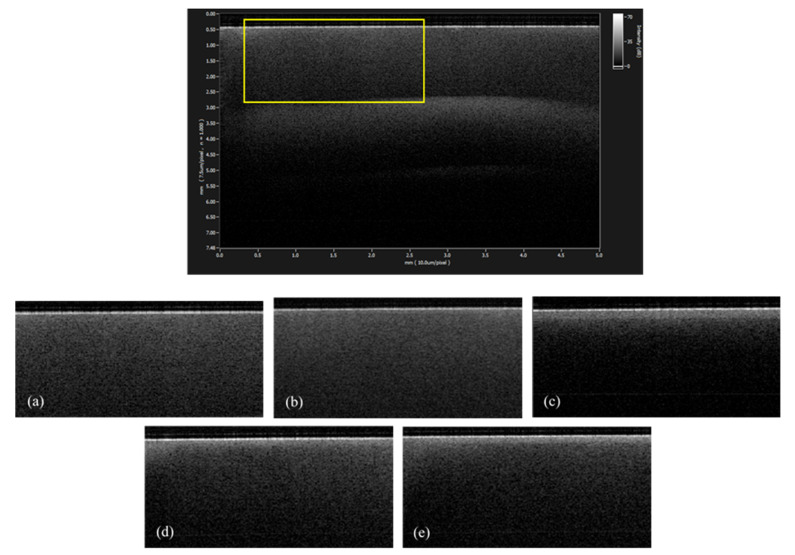
Representative SS-OCT images in each group. The whole image (1) and part surrounded by yellow, including the dentin surface (2). In each group, (2) was adopted as an evaluation target. The dentin surface of the group 3 (**c**) appeared clearly distinct from that of the group 1 (**a**). The images of the group 4 (**d**) and group 5 (**e**) showed similar brightness of the dentin surface as that of the group 1 (**a**) and group 2 (**b**). (**a**) group 1, sound, (**b**) group 2, de3, (**c**) group 3, de6, (**d**) group 4, CS, and (**e**) group 5, NS.

The SS-OCT images obtained from each group are presented in Figure 3. On the reconstructed gray-scale image, the dentin surface after six days of demineralization appeared clearly distinct from the sound dentin with increased brightness. The images of CS and NS groups were characterized by similar brightness of the dentin surface with sound or de3 groups.

**Figure 4:**
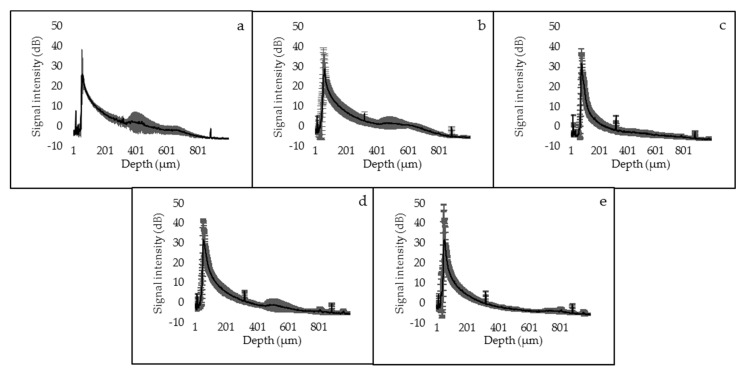
Signal intensity profile of each group up to 800 µm depth. The data were analyzed based on the observation results of 25 points in each group observed by SS-OCT, and a signal intensity profile of each group was created. The signal intensity of the sound group (**a**) and de3 group (**b**) suddenly dropped in the deeper parts. The signal profiles of the CS group (**d**) and NS group (**e**) showed intermediate patterns between those of the sound group (**a**) and de6 group (**c**) starting at high intensity and gradually decreasing. (**a**) Sound group, (**b**) de3 group, (**c**) de6 group, (**d**) CS group, and (**e**) NS group.

**Figure 5:**
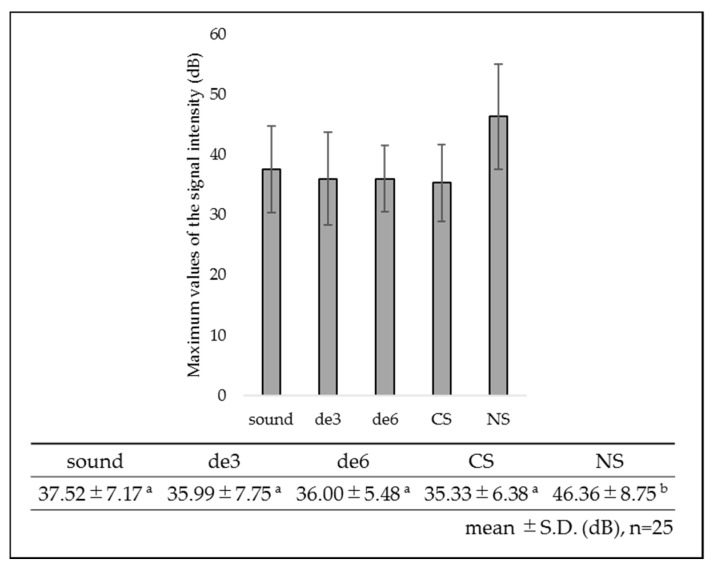
Maximum values of signal intensity (dB) for each group. NS showed the highest maximum value of signal intensity within the groups. The same lowercase letters indicate no significant differences among the values (*p* > 0.05).

The SS-OCT signal profile and maximum values of signal intensity in dB unit obtained from the dentin surfaces are shown in Figure 4 and Figure 5. The sound and de3 groups showed similar SS-OCT signal profiles and maximum values of signal intensity. In de6 group, although the signal profile from the superficial layer showed nearly the same intensity level as that of the sound dentin or de3 groups, the signal intensity suddenly dropped in the deeper parts. The signal profiles of CS and NS groups showed intermediate patterns between those of the sound and de6 group starting at high intensity and gradually decreasing. NS group showed the highest maximum value of the signal intensity within the groups.

**Figure 6:**
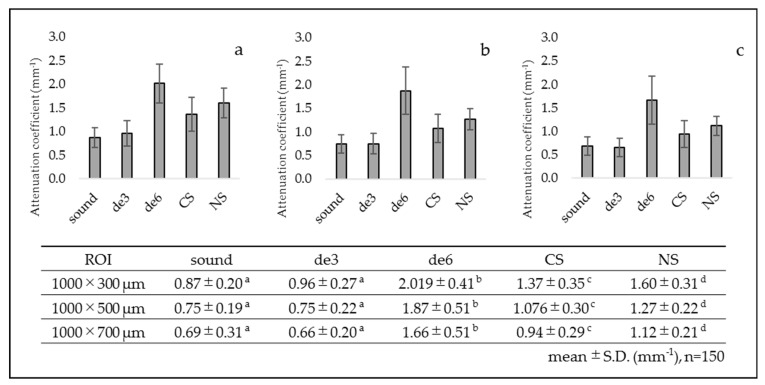
Integrated signal intensity in each ROI. CS and NS showed higher values than de6 for all the ROI depths (*p* < 0.05). (**a**) ROI 1000 × 300 µm ^2^, (**b**) ROI 1000 × 500 µm^2^, and (**c**) ROI 1000 × 700 µm^2^. The same lowercase letters indicate no significant differences among the values (*p* > 0.05). (One-way ANOVA with Tukey’s post hoc).

The results of integrated signal intensity obtained from the ROI of 300, 500, and 700 µm in depth are presented in Figure 6. For de6 group, the integrated signal intensity from 300 µm in depth showed no significant difference from the sound group. However, the values of 500 and 700 µm in depth were significantly lower than those of the other groups. Conversely, the values of de3 group showed higher results than the sound group. The results of CS and NS groups showed higher integrated values than those of the de6 group for all the ROI depths.

**Figure 7:**
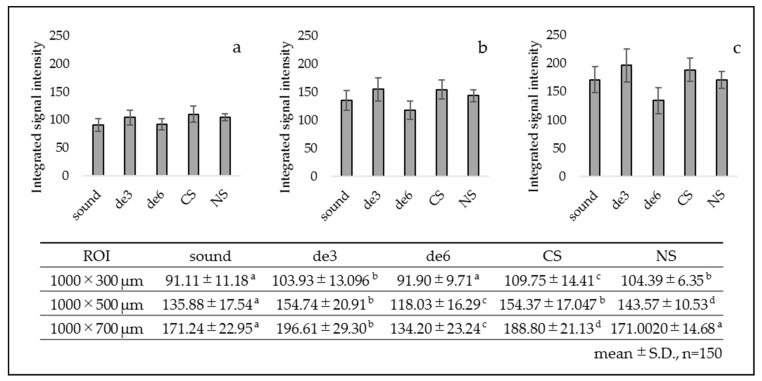
Attenuation coefficient (µt) in each ROI. CS and NS showed intermediate values between the sound and de6. (**a**) ROI 1000 × 300 µm^2^, (**b**) ROI 1000 × 500 µm^2^, and (**c**) ROI 1000 × 700 µm^2^. The same lowercase letters indicate no significant differences among the values (*p* > 0.05). (One-way ANOVA with Tukey’s post hoc).

The results of attenuation coefficient obtained from each ROI are presented in Figure 7. Overall, de3 group showed nearly the same values as the sound group for all the ROIs. In contrast, de6 group showed significantly higher attenuation coefficient than the other groups for all the ROIs. CS and NS groups showed intermediate values of attenuation coefficient between the sound and de6 groups.

### 3.2. SEM Analysis

**Figure 8:**
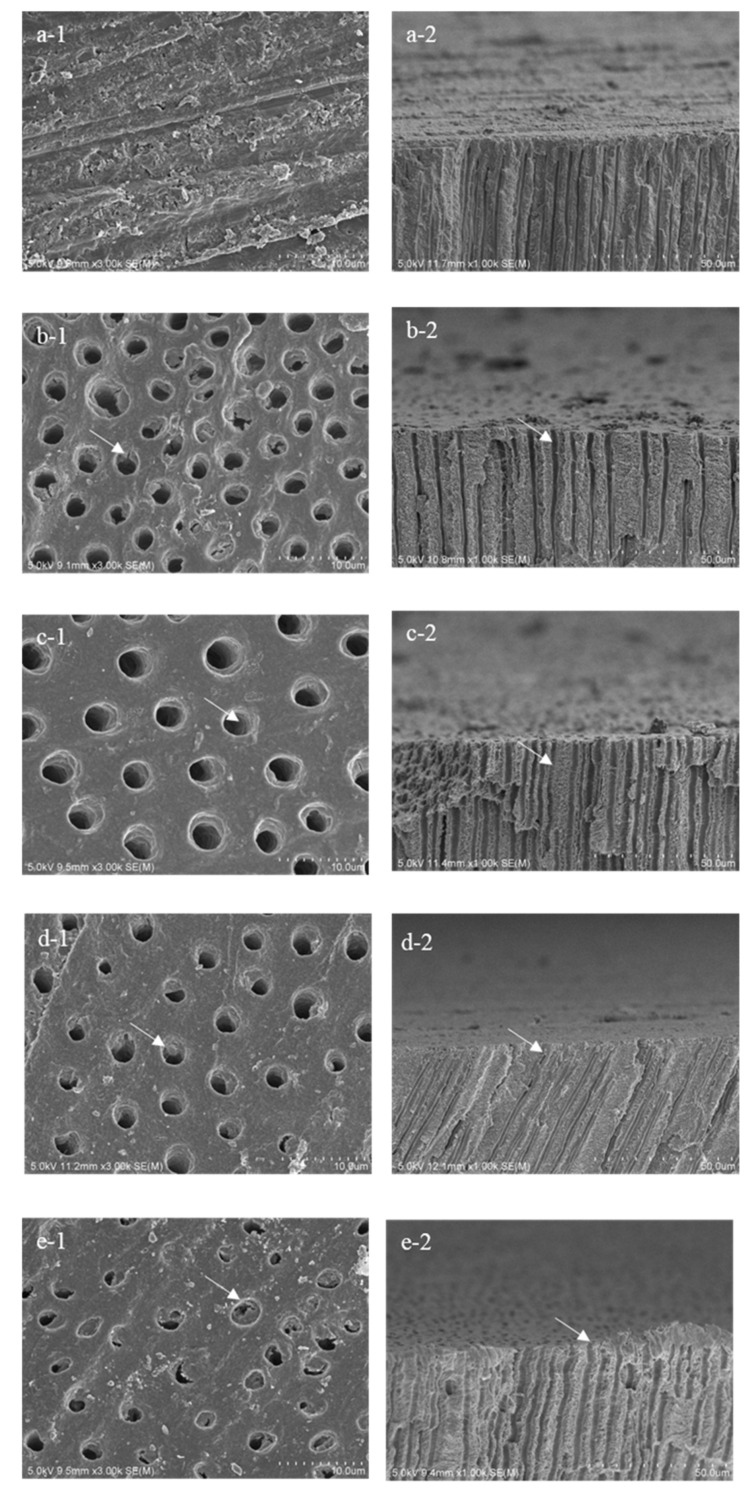
SEM images of the dentin surface (1) and sagittally fractured surface (2) of each group. In the de3 group (**b**), the morphological structure of the peritubular dentin remained (arrow). The peritubular dentin lost its morphological structure in the de6 group (**c**, arrow). In the CS group (**d**), remnants were detected within the dentinal tubules (arrow). Reactional deposits were observed on the dentin surface in the NS group (**e**, arrow). (**a**) Sound group, (**b**) de3 group, (**c**) de6 group, (**d**) CS group, and (**e**) NS group.

**Figure 9:**
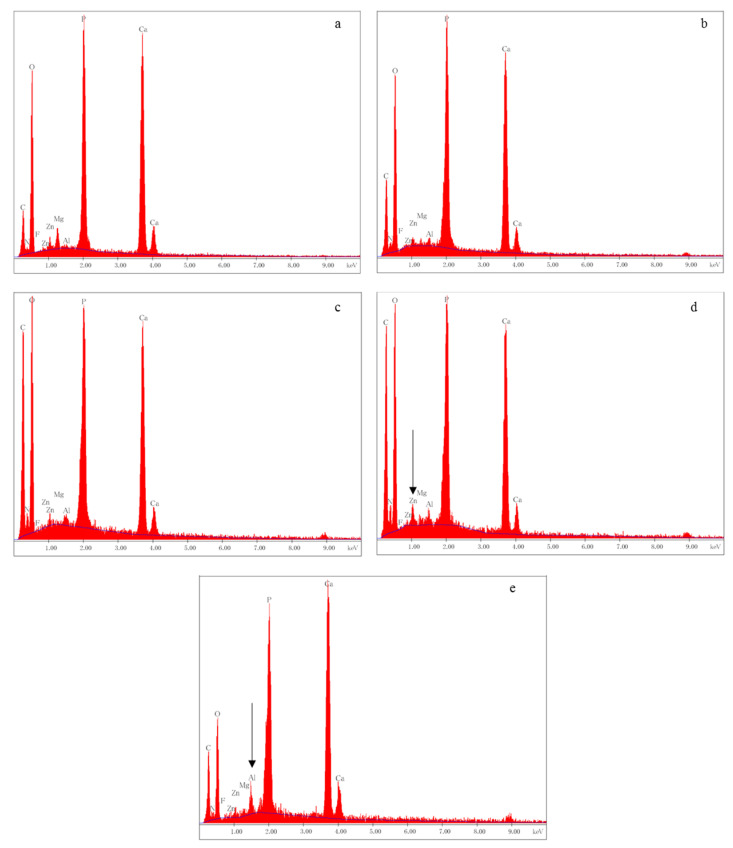
EDX spectra of sagittally fractured surfaces of each group. In the CS group (**d**), zinc was detected (arrow). In the NS group (**e**), aluminum was detected (arrow). (**a**) Sound group, (**b**) de3 group, (**c**) de6 group, (**d**) CS group, and (**e**) NS group.

Representative SEM images and EDX spectra obtained from the sagittally fractured dentin surfaces are shown in Figure 8 and Figure 9, respectively. The surface of the sound group was covered with a smear layer, and the dentinal tubules were occluded by the smear plugs (Figure 8(a-1)).

The three days of demineralization in this study (de3 group) removed the smear and opened the dentinal tubules, with slight demineralization of superficial intertubular dentin. However, the morphological structure of peritubular dentin appeared to remain (Figure 8(b-1,b-2)).

After six days of demineralization, the orifice of dentinal tubules was enlarged in diameter because of the further demineralization. The intertubular dentin of de6 group was demineralized deeper than the de3 group, with the loss of morphological structure of peritubular dentin. (Figure 8(c-1,c-2)). The SEM image of the dentin surface in Figure 8(d-1) showed some remnants of reactional deposits on the dentin surface in CS group, even after the additional three days of demineralization. In NS group, although the reactional deposits did not remain after the additional three days of demineralization, the morphological structure of the peritubular dentin clearly remained (Figure 8(d-2)).

The results of EDX analysis obtained from the groups are presented in Figure 9. In CS group, zinc was detected from the sagittally fractured dentin surface (Figure 9d), whereas aluminum was detected in NS group (Figure 9e).

## 4. Discussion

In this study, artificial demineralization of the dentin surface was achieved by immersing dentin discs for three days (de3) and six days (de6) in a demineralization solution adjusted to pH 5.0. Erosion has been considered the main etiological factor for DH [9,32,39]. Therefore, three days of demineralization was used in this study to create the erosive dentin with the opening of dentinal tubules. The results of SEM analysis confirmed that after three days of demineralization, the dentin surface showed slight demineralization with opened dentinal tubules (Figure 8). The inhibitory effect of desensitizers against further demineralization was evaluated by the analysis of OCT signal after the additional three days of demineralization. The variation of the optical property because of the mineral loss was determined by evaluating the maximum value of the signal intensity, integrated signal, and attenuation coefficient of the OCT signal. In the comparison of OCT signal with that of previous studies, Mona et al. reported that the attenuation coefficient for sound dentin was 1.05 ± 0.3 in ROI that was 200 µm in width with a 400 µm depth [36], whereas Wada et al. showed that the values for sound dentin and naturally demineralized dentin were 0.84 ± 0.36 and 1.62 ± 0.59, respectively, with the ROI for 5000 µm in width and 500 µm in depth [40]. In the current study, ROIs of three depths of 300, 500, and 700 µm with a constant width of 1000 µm were employed. The attenuation coefficient of sound dentin for the depths of 300, 500, and 700 µm were 0.87 ± 0.20, 0.75 ± 0.19, and 0.68 ± 0.19, respectively. In addition, the OCT signal profile of sound dentin in this study was similar to that reported in previous studies, with a decreasing trend of the attenuation coefficient with increasing ROI depth.

In this study, OCT signal after three days of demineralization presented a similar profile as that of sound dentin. No significant differences of attenuation coefficient or maximum value of the signal intensity were observed between the values of sound dentin and three days demineralization (*p* > 0.05). However, integrated OCT signals after three days of demineralization were significantly higher than those of the sound dentin for all the ROI depths (*p* < 0.05). Because backscattered OCT signal from the structure was obtained between the media with different refractive indices, the intensified signal after the three days of demineralization most likely arose from the mineral loss, forming dentin porosity, producing micro-interfaces, and resulting in higher reflectivity and increased brightness [41].

The OCT signal profile after six days of demineralization revealed clear reduction of signal intensity toward deeper dentin, resulting in a higher attenuation coefficient than after three days of demineralization for all the ROI depths (*p* < 0.05). Moreover, the values of the integrated signal intensity after six days of demineralization were significantly lower than those of sound dentin and after three days demineralization for the ROIs of 500 and 700 µm (*p* < 0.05) [42]. The highly demineralized dentin appeared to contain fewer minerals, leaving organic dentin matrix. Although collagen is optically nonlinear and known to scatter light, gelatinized or fragment collagen is considered less scattering [26,43]. Consequently, six days of demineralization appears too aggressive, affecting the optical property of the substrate, resulting in less scattering than the three days of demineralization.

In this study, the maximum signal intensity of SS-OCT obtained from NS group was significantly higher than those of the other groups (*p* < 0.05). NS forms a nanoparticle-sized layer as the reactional product comprising CaF_2_, Ca_3_(PO_4_), phosphate silicate, and fluoro-alumino-silicate glass, with a thickness of approximately 1 µm on the dentin surface [16,44]. Although nanoparticle deposits of NS group were less soluble even in the acidic solution, the enhanced maximum value of signal intensity of NS group from the superficial zone was likely because of the scattering from the nanoparticle deposits remaining on the dentin surface even after the additional three days of demineralization [14]. The results of our SEM study correlate well with previous studies and showed that the reactional products remained on the dentin surface even after the additional three days of demineralization (Figure 8(e-1,2)).

MMPs are involved in collagen degradation of caries’ process [45]. When the pH is lowered and the dentin is demineralized, MMPs are activated and expose the organic matrix [46]. Zinc strongly reduces MMP-mediated collagen degradation in partially demineralized dentin [20,21,47]. In this study, the EDX analysis revealed the presence of zinc from the sagittally fractured dentin surface in the CS group (Figure 9d). Liu et al. reported that the release of cationic Zn^2+^ and Ca^2+^ from CS was accelerated under the acidic conditions of pH 4.5 and 5.0 [42]. In the current study, an acetic acid-based solution with pH 5.0 was employed as the artificial demineralization solution, and the solution was replaced every day during the process. The condition of the demineralization solution in this study created an environment in which zinc was easily released from CS applied to the dentin. The zinc increased the potential for intrafibrillar remineralization at partially demineralized collagen matrices, favoring dentin remineralization [48]. Zinc released from the CS binds to calcium and phosphate in the teeth and crystallizes to form a nanoparticle-sized layer on the dentin. In this study, most of the deposits on the dentin surface disappeared after an additional three days of demineralization (Figure 8(d-1)). In the CS group, the maximum signal intensity of SS-OCT was less than that in the NS group (Figure 5) because of the disappearance of deposits on the dentin surface. The deposits on the dentin surface in the CS group were considered to have disappeared upon immersion in the demineralization solution at pH 5.0 for three days. It is necessary for the formation of new mineral that the pre-existing crystal seeds serve as nucleation sites [49]. The intertubular dentin is rich in collagen; therefore, during the three-day demineralization, the pre-existing crystal seeds were reduced and the scaffolding for the formation of new mineral may have been reduced. However, even after the additional three days of demineralization, the deposits of CS remained on the lumen of the dentinal tubules (Figure 8(d-1,2)). Toledano et al. applied zinc-containing polymer nanogels to the demineralized dentin (demineralization solution:citric acid solution, pH 3.8) for 1 min, evaluated after seven days, and reported high nanohardness and high remineralization on the peritubular dentin. Many crystal seeds were present in the peritubular dentin because of the hypermineralization [21]. Consequently, deposits containing Zn remained on the lumen of the dentinal tubules even after additional demineralization. Zinc released from the CS can easily remain in areas that are originally rich in dentin minerals. Inhibition of MMP activity by zinc may maintain the structure of dentin and induce demineralization inhibition effects of CS.

OCT signal of CS group sample showed significantly higher integrated values and lower attenuation coefficients from the ROIs of 300, 500, and 700 µm than those of the NS group samples. The CS group showed less discrepancy of this optical value compared with the sound dentin because both the CS and NS groups showed intermediate values of the attenuation coefficient between the sound dentin and six-days demineralized dentin, suggesting the preventive effect against further demineralization.

Some deposits remained under the surface rather than on the surface of the dentinal tubules in the CS group, as observed in the SEM images. For the sagittally fractured surface, deposits were observed on the inner wall of the dentine tubules (Figure 8). This result appears to suggest the demineralization inhibition effects of CS on the inner wall of the dentine tubules.

The current in vitro study highlights the ability of dentin desensitizers to withstand the challenges of demineralization. Because of the limitations of reactional products of dentin desensitizers against chemical and mechanical challenges, the results emphasize that the effects of the desensitizers on the dentin surface to protect against the acidic challenges were limited and could be observed within the additional three days of demineralization. Clinically, the oral cavity is a dynamic environment because of the presence of occlusions, mastication, and tooth-brushing abrasion. Although long-lasting desensitizing effect or anti-demineralization effect of the desensitizers appears difficult to achieve, dentin desensitizers represent a less invasive treatment and their application can be repeated. Further studies should be conducted to confirm the long-term effects of these products against the high frequency of acidic beverage consumption and tooth brushing.

## 5. Conclusions

Under the conditions and limitations of this in vitro study, both dentin desensitizers for Caredyne Shield and Nanoseal showed inhibition effects against demineralization from different mechanisms of action. Our findings demonstrate that SS-OCT signal analysis can be used for monitoring dentin demineralization after the application of these materials in vitro.

## Figures and Tables

**Table 1:** Compositions and application methods of the desensitizers.

Material	Manufacturer	Composition	Method of Application	Lot No.
Caredyne Shield (CS)	GC, Tokyo, Japan	Liquid A: Fluoro-zinc silicate glass Liquid B: Phosphoric acid	Liquid A and B are mixed equally and apply to the dentin surface for 5 to 20 s and rinse with water.	1804121
Nanoseal (NS)	Nippon Shika Yakuhin, Yamaguchi, Japan	Liquid A: Fluoro-alumino-calcium silicate glass Liquid B: Phosphoric acid		J1P

## Data Availability

Not applicable.
